# Knowledge and perception of COVID-19 vaccination in two districts of Yaoundé

**DOI:** 10.4102/jphia.v16i1.860

**Published:** 2025-04-07

**Authors:** Michelle S. Djuidje Kamguia, Yves Le Grand Napa Tchuedji, Albert Ze

**Affiliations:** 1 Higher School of Health Sciences Rosière, Yaoundé, Cameroon; 2 Association for Disease Surveillance and Social Assistance, Yaoundé, Cameroon; 3 Department of Health Sciences, Association for Disease Surveillance and Social Assistance, Yaoundé, Cameroon; 4 Department of Microbiology, Faculty of Science, University of Yaoundé I, Yaoundé, Cameroon; 5 Research Institute for Health and Development, Yaoundé, Cameroon

**Keywords:** perception, knowledge, vaccination, COVID-19, health districts, SARS-CoV-2, Nkolndongo, Cité verte

## Abstract

**Background:**

The coronavirus disease 2019 (COVID-19) pandemic has caused considerable problems throughout the world, with an impact on both public health and economic development. The rapid spread of severe acute respiratory syndrome coronavirus 2, has led researchers to progressively develop vaccines.

**Aim:**

This study aimed to assess the perceptions and knowledge of the population of two health districts in Cameroon about vaccination against COVID-19.

**Setting:**

This study was conducted in Cameroon from November 2021 to July 2022, targeting any Cameroonian citizen over 18 years residing in Yaoundé.

**Methods:**

We conducted a cross-sectional study in two health districts of Yaoundé (Nkolndongo and Cité verte) targeting residents of each selected district who were over 18 years. The minimum sample size was calculated using the vaccination coverage rate in each district. The sampling was systematic and the questionnaire included knowledge and perceptions about the COVID-19 vaccine. The data were processed using Graphpad 8.0.1 and Microsoft Excel 2016.

**Results:**

Of the 100 participants, 59% were women. Ninety per cent of the participants had not been vaccinated against COVID-19 and 80% of those vaccinated had received one dose of the vaccine. Participants showed poor adherence to the COVID-19 vaccine, except for hairdressers (*p* = 0.158) and students from secondary education (*p* = 0.158). Vaccination was the least known preventive method (3%). Most people were obliged to take the vaccine (80%) and most religions (75%) were associated with vaccine refusal (*p* ≤ 0.0005).

**Conclusion:**

Knowledge and perceptions of COVID-19 vaccination were low and were influenced by the socio-cultural environment. It is necessary to develop a national policy for the promotion of vaccination adapted to the socio-cultural environment when planning the introduction of a vaccine.

**Contribution:**

This study demonstrates the importance of socio -anthropological, religious and scientific indicators during the planification of introduction of new vaccine in the event of pandemic or new outbreak.

## Introduction

Coronavirus disease 2019 (COVID-19) is defined as a disease caused by the severe acute respiratory syndrome coronavirus 2 (SARS-CoV-2). The number of deaths because of COVID-19 demonstrates that all countries should invest in more resilient health systems that can sustain essential health services during crises.^1^ The first case of COVID-19 in Cameroon was detected in the city of Yaoundé in March 2020,^2^ and cases spread rapidly to other cities. The World Health Organization (WHO) shows that the death toll associated with the COVID-19 pandemic between 2020 and 2021 was approximately 14.9 million.^1^ In Cameroon there were 1915 deaths because of COVID-19.^3^ Yaoundé was one of the cities in Cameroon with the highest number of confirmed cases and deaths. Several measures have been taken to combat this pandemic in Cameroon, including therapeutic protocols based on chloroquine, vitamin C and zinc, herbal treatments and the administration of vaccines. The overall objective of introducing the COVID-19 vaccine in Cameroon was to vaccinate at least 80% of vulnerable populations by December 2021, approximately 14 million people,^4^ with the aim to contain the pandemic.^5^ However, the typical experiences associated with vaccines were not immediately observed with the COVID-19 vaccines. Surveys that emerged from various contexts indicated widespread hesitancy about vaccines against COVID-19. According to the WHO, 22 African countries used less than a quarter of the vaccines supplied to them.^6^ In Cameroon, the results of vaccination coverage in December 2021 revealed low coverage rates for the 10 regions, with values of 5.9% and 4.7% for doses 1 and 2, respectively. Given these low coverage rates, we decided to assess the knowledge and perceptions of the population in the districts of Nkolndongo and Cité verte about vaccination against COVID-19 to identify action indicators that will enable better vaccination coverage to be achieved in the event of a pandemic as a result of an emerging disease in the future.

## Research methods and design

### Study setting

This was a cross-sectional study, conducted in Cameroon from November 2021 to July 2022, targeting any Cameroonian citizen aged 18 years and older residing in Yaoundé. Participants were given an anonymous bilingual (French, English) questionnaire that has been validated by the research team. The sampling method was random. Data were recorded using Microsoft Excel 2016 and analysed with Graphpad 8.01.

### Study population

This study targeted populations of Nkolndongo and Cité verte districts. The District of Cité verte reported about 419 995 inhabitants with an immunisation coverage rate of 1.9% and the District of Nkolndongo reported about 573 031 inhabitants with an immunisation coverage rate of 0.6% in 2022, based on the 2022 district data. The source included persons aged 18 years and older residing in the Nkolndongo and Cité verte districts at the time of the study. The sample size was determined using the formula (see Equation 1) described by Cochran^7^:


$$N=t^{2}.\text{P}.\text{Q}/d^{2}$$ [Eqn 1]

with *N*: the minimum sample size, *t*: 1.96, P: immunisation coverage rate (1.9% in Cité verte and 0.6% in Nkolndongo), Q: 1-P, *d*: margin error (0.05). As the sample size was small in each of the districts (< 30), we decided to increase the sample size to 58 participants in the Nkolndongo health district, which had the highest proportion of residents, and to 42 participants in the Cité verte health district. Clear information was given about the study, informed consent was obtained from all participants. Adults over 18 years, both males and females, from different educational levels and who responded to the article questionnaire were included. Exclusion criteria included incorrectly filled-in information.

### Data collection

A paper questionnaire was tested on five people who had information about the study and then on the members of the research team. Corrections were made to ensure that all possible answers were included, and then the questionnaire was validated by the work supervisor. Socio-demographic data, data on knowledge and perceptions of COVID-19 were collected.

### Procedures

Participants were selected randomly in each district. The collection sites were selected from a sampling frame listing all neighbourhoods. Five neighbourhoods were selected randomly in each district based on a list of neighbourhoods of each district: Briqueterie, Yoyo bar, Tsinga, Carrière and Mokolo in Cité verte and Odza, Awae, Terminus Mimboman, Kondengui and Ekie in Nkolndongo. In each neighbourhood, a crossroads represented a reference point. From each reference point, a street was chosen and data collection began at the first household (*n*_0_). The households were selected progressively after a two-step order (*n*_0_ + 2). Data collection was conducted from November 2021 to July 2022. Data were collected using a validated questionnaire covering: socio-demographic characteristics (profession, religion, gender), perception (vaccine adherence, vaccine preferences) and knowledge (prevention method against COVID-19).

### Data analysis

All the data collected were recorded and organised into an Excel spreadsheet. The data were analysed with Graphpad 8.01. Data were presented in tables. Each qualitative variable were expressed as effective or frequencies. Student’s *t*-test was performed to compare characteristics of each variable and *p*-value < 0.05 was considered statistically significant. We organised our analysis into three parts. Firstly, we presented a description of the population, secondly an overview of perceptions about COVID-19 and thirdly a knowledge about COVID-19 preventive method.

### Ethical considerations

This work was submitted to the Centre’s Regional Ethics Committee for Human Health Research and ethical clearance was obtained under the number CE N0 N256/CRERSHC/2022. This evaluation was conducted according to the guidelines of the Declaration of Helsinki. Written consent of participants was obtained, the data of participants were anonymised before being used and all precautions were taken to avoid any risk of patient identification.

## Results

A total of 100 participants were included in the study. Women (59%) were the most represented. The highest frequency of participants (38%) were aged between 18 and 28 years, with a minimum age of 18 years and a maximum age of 88 years.

### Distribution of participants according to vaccine adherence

In this study, 90 of the 100 participants were not vaccinated against COVID-19. Of those vaccinated, 80% had received one dose of the vaccine and 20% had received two doses. Sixty per cent of those vaccinated were from the Nkolndongo district and 40% from the Cité verte district. For all professions, most participants did not take the vaccine, except for hairdressers, who did not show a statistically significant difference between vaccinated and non-vaccinated participants (Table 1). There was an association between religion and low adherence to the COVID-19 vaccine (*p* < 0.05), except for participants from the Revivalist church, where the difference was not significant. In terms of gender, most participants were unvaccinated, whether they were men or women.

**TABLE 1:** Factors associated with vaccine adherence.

Variables	Unvaccinated (%)	Vaccinated (%)	*p*-value	95% CI
**Profession**				
Hairdresser	1	0	0.1583	−2.603 to 0.6030
Trader	11	1	< 0.0001	−11.60 to −8.397
Farmer	3	0	0.0065	−4.603 to −1.397
Student from secondary education	3	0	0.0065	−4.603 to −1.397
Students from higher education	22	0	< 00001	−23.60 to −20.40
Government employee	19	4	< 0.0001	−16.60 to −13.40
Health care personnel	12	1	< 0.0001	−12.60 to −9.397
Others	15	3	< 0.0001	−13.60 to −10.40
Unemployed	4	1	0.0065	−4.603 to −1.397
**Religion**				
Catholic	52	6	< 0.0001	−47.60 to −44.40
Muslim	6	0	0.0005	−7.603 to −4.397
Protestants	30	3	< 0.0001	−28.60 to −25.40
Revival churches	2	1	0.1583	−2.603 to 0.6030
**Gender**				
Male	35	6	< 0.0001	−30.60 to −27.40
Female	55	4	< 0.0001	−52.60 to −49.40

### Motivation for refusing or accepting the vaccine

Coronavirus disease 2019 vaccination was on the one hand forced, with 80% of those vaccinated stating that the vaccine constituted a health pass for access to certain environments, although 20% of those vaccinated stated that they took the vaccine voluntarily to improve their health at an individual and community level (Table 2). The reasons for vaccine refusal were diverse. Some individuals viewed the vaccines as a scheme by large organisations to exert control over the world (28.9%), while others expressed disinterest (26.7%), or concerns about the reliability of the vaccines, fearing potential complications (18.9%). In addition, 11.1% of unvaccinated individuals believed that maintaining a healthy lifestyle was sufficient to prevent the disease.

**TABLE 2:** Distribution of participants according to their motivation for refusing or accepting the vaccine.

Motivations	Effective	Frequency (%)
**Reasons for acceptance (*N* = 10)**		
Vaccines help to improve people’s health	2	20.0
Vaccines are used as health pass in some environments	8	80.0
**Reason for refusal (*N* = 90)**		
The vaccine contains particles that will control my brain	4	4.4
The vaccine against COVID-19 is part of the schemes of a major company which created the disease to control the world	26	28.9
The vaccines available to us are those rejected by developed countries and which cause many complications	17	18.9
A healthy lifestyle helps prevent this disease	10	11.1
Not interested	24	26.7
I have a natural immunity because I am constantly in contact with the virus	1	1.1
Taking the vaccine does not prevent	1	1.1
The vaccine contains adverse health effects	1	1.1
A lack of awareness	1	1.1
Vaccines are useless	5	5.6

COVID-19, coronavirus disease 2019.

### Vaccine preferences

Vaccinated participants had a strong preference for the Jonhson and Jonhson vaccine (50%), followed by the AstraZeneca vaccine (30%) and the Sinopharm vaccine (10%). However, 10% of participants did not know which vaccine they had received (Table 3).

**TABLE 3:** Distribution of participants according to vaccine types.

Vaccine types	Effective	Frequency (%)
Sinopharm	1	10
AstraZeneca	3	30
Jonhson and Jonhson	5	50
Unknown	1	10

### Participants’ knowledge of ways to prevent coronavirus disease 2019

Assessment of participants’ knowledge of measures to prevent COVID-19 (Table 4), revealed that the main preventive methods known were barrier measures (73%) and traditional medicines (13%). Vaccination was the least known preventive method. A low adherence to the COVID-19 vaccine was observed regardless of the level of knowledge of preventive methods.

**TABLE 4:** Knowledge of preventive methods against coronavirus disease 2019 and vaccine adherence.

Prevention methods	Frequency (%)	Unvaccinated (*n*)	Vaccinated (*n*)	*p*-value	95% CI
Barrier measures	73	66	7	< 0.0001	−60.60 to −57.40
Traditional drugs	13	12	1	< 0.0001	−12.60 to −9.397
Vaccination	3	2	1	0.1583	−2.603 to 0.6030
Barrier measures, traditional drugs	5	5	0	0.0010	−6.603 to −3.397
Barrier measures, traditional drugs and vaccination	5	4	1	0.0065	−4.603 to −1.397
Barrier measures and vaccination	1	1	0	0.1583	−0.6030 to 2.603

**Total**	**100**	**-**	**-**	**-**	**-**

## Discussion

Coronavirus disease 2019 was one of the most important pandemics in recent years in terms of its incidence rate, the number of deaths and the economic losses caused worldwide. The measures put in place to control the epidemic were based on barrier measures, awareness-raising, vaccination and medicinal and traditional treatments. However, the knowledge and perceptions of the population were indicators that had an impact on the evolution of the epidemic. In this study, we assessed the knowledge and perceptions of the population in two districts of Cameroon regarding vaccination against COVID-19.

The majority of participants were young adults, although older adults were also represented. Studies conducted by Sako,^8^ in Mali also showed that young adults were the most represented. In our study, only 20% of participants used the COVID-19 vaccine, with 80% taking a single dose. This may be explained by low public awareness, misinformation and doubts about the reliability of these vaccines. These results show that the promotion of a new vaccine as a preventive method must be well-planned, especially in an emergency situation, and its implementation must take account of socio-anthropological realities.

The use of new vaccines in emergencies is very difficult in Cameroon. Apart from hairdressers and students from secondary schools, all other professions refused the vaccine. The involvement of health workers and civil servants among those refusing the COVID-19 vaccine shows that there are also scientific concerns, although some participants are prejudiced. This is the case of some participants who stated that ‘The vaccine against COVID-19 is part of the schemes of a big company that created the disease to control the world’ and others who stated ‘The vaccines available to us are those rejected by Westerners and which give many complications’. The position taken by health professionals may explain why a large proportion of the population did not adhere to the vaccine, given that the opinions of health professionals have a considerable impact on the population. Studies conducted by Julio et al. revealed that health workers are the most reliable sources of advice on COVID-19 vaccines.^9^ Our results differed from those of the study conducted by Julio et al. who found that the most common reason given for vaccine acceptance was personal protection against SARS-CoV-2 infection.^9^ Vaccine hesitancy, also known as anti-vax or anti-vaccination, has been identified by the WHO as one of the top 10 global health threats.^10^

The role of religious beliefs in vaccine refusal should not be overlooked, as Protestant, Catholic and Muslim participants were significantly associated with refusal of the COVID-19 vaccine. This may be explained by negative campaigns by certain religious leaders who did not believe in the real existence of the disease, leading to the closure of certain churches by the Cameroon authorities.

Although a minority of participants had taken the vaccine, 80% said they had done so to access services that required it. This situation shows that the population’s perceptions of the COVID-19 vaccine are negative overall. These results are similar to a previous study, which revealed that people consider vaccines against COVID-19 to be less safe and ineffective than other vaccines.^6^ The most popular vaccines were the Johnson & Johnson vaccine (50%) and the AstraZeneca vaccine (30%). Previous studies conducted in Canada have also revealed that hesitancy towards the AstraZeneca vaccine also appears to exceed that towards Johnson & Johnson.^11^

However, concerns remain about these two vaccines, as a previous study conducted in Canada showed that the intention, perceived efficacy and safety of Canadians were, respectively, 0.14 points, 0.11 points and 0.11 points lower on a 0 to 1 scale for AstraZeneca and Johnson & Johnson than for Pfizer and Moderna.^11^

In addition to perception, it should be noticed that a good proportion knew about barrier measures and traditional drugs as preventive methods against COVID-19, while only 3% knew that vaccination is a preventive method against COVID-19. Previous studies have also revealed the consumption of herbal drinks, to prevent COVID-19.^7^ In contrast to our results, the study by Al-Zalfawi et al., in Saudi Arabia found that participants aged 18–59 years showed better knowledge, attitude and perception towards COVID-19 vaccination.^12^

This study revealed that various indicators are associated with the refusal of the COVID-19 vaccine. These include scientific reasons, religious perceptions, misinformation, a lack of knowledge and cultural perceptions. These indicators should make it possible to understand that the implementation of a new vaccine must be well-planned, and that good promotion and strong awareness-raising must be considered in advance, taking account of the socio-anthropological environment.

### Limitations

Our study has some limitations, notably the small sample size, which could result in insufficient power to detect significant differences in the analyses. It is also important to carry out the study in several districts of Cameroon and other countries.

## Conclusion

This study revealed that people’s knowledge and perceptions of vaccination against COVID-19 are not good and are the cause of low adherence to the COVID-19 vaccine in Cameroon. It is important to consider socio-anthropological, religious and scientific indicators when planning the introduction of a new vaccine in the event of a pandemic or a new outbreak.
